# Predicting the Features of Methane Adsorption in Large Pore Metal-Organic Frameworks for Energy Storage

**DOI:** 10.3390/nano8100818

**Published:** 2018-10-11

**Authors:** George Manos, Lawrence J. Dunne

**Affiliations:** 1 Department of Chemical Engineering, University College London, Torrington Place, London WC1E 7JE, UK; g.manos@ucl.ac.uk; 2 School of Engineering, London South Bank University, London SE1 0AA, UK; 3 Department of Chemistry, University of Sussex, Falmer, Brighton BN1 9QJ, UK

**Keywords:** metal–organic framework, negative gas adsorption (NGA), osmotic ensemble (OE), mechanical pressure, methane storage

## Abstract

Currently, metal-organic frameworks (MOFs) are receiving significant attention as part of an international push to use their special properties in an extensive variety of energy applications. In particular, MOFs have exceptional potential for gas storage especially for methane and hydrogen for automobiles. However, using theoretical approaches to investigate this important problem presents various difficulties. Here we present the outcomes of a basic theoretical investigation into methane adsorption in large pore MOFs with the aim of capturing the unique features of this phenomenon. We have developed a pseudo one-dimensional statistical mechanical theory of adsorption of gas in a MOF with both narrow and large pores, which is solved exactly using a transfer matrix technique in the Osmotic Ensemble (OE). The theory effectively describes the distinctive features of adsorption of gas isotherms in MOFs. The characteristic forms of adsorption isotherms in MOFs reflect changes in structure caused by adsorption of gas and compressive stress. Of extraordinary importance for gas storage for energy applications, we find two regimes of Negative gas adsorption (NGA) where gas pressure causes the MOF to transform from the large pore to the narrow pore structure. These transformations can be induced by mechanical compression and conceivably used in an engine to discharge adsorbed gas from the MOF. The elements which govern NGA in MOFs with large pores are identified. Our study may help guide the difficult program of work for computer simulation studies of gas storage in MOFs with large pores.

## 1. Introduction

In the next few years, a revolution is expected to occur in energy storage that will modify the way energy is used and the impact it has on climate change [1,2]. The demand for energy storage is rising rapidly as it is a key factor in the development of clean renewable energy technologies. New cleaner electric vehicle technologies are replacing old dirty combustion engines. Yet, challenging problems remain. The bridge between transportation using dirty combustion fossil fuel engines and more versatile and cleaner newer designs is the focus of intense research endeavours, but the main problem remains the distance which electric automobiles can cover by battery power alone. 

Storage of energy carriers (H_2_, CH_4_) in absorbent materials is a very promising innovative energy solution [3,4] enhancing the driving range of hybrid electric automobiles. Metal-organic frameworks (MOFs) are such absorbent materials which have become the focus of an international endeavour to utilize their unique properties in an extensive range of energy applications [5,6,7,8,9]. In particular, large pore MOFs are likely to be the best candidates for gas storage for energy applications. Yet new and unexplored features and properties of these MOFs appear with increasing pore-size. To be quite clear, these novel features appear when the change in volume in the narrow to large pore transformation is such that the free energy contributions from the volume change become dominant. The numerical values involved depend upon the pressure regime under consideration. As is shown in detail below, with pore sizes and volumes explicitly given, low pressure features require large pore volume changes and vice versa.

MOFs are hybrid organic-inorganic nanoporous materials with remarkable adsorption properties in which organic units link metal framework centres which afford structural flexibility, thereby allowing transformations to occur between different pore systems. They make up a large class of soft absorbent materials, designated by Kitagawa and co-workers [10] as “absorbent crystals that possess both a highly ordered network and structural transformability”. MOFs have high potential for use in adsorptive separation processes [11,12,13,14,15,16,17,18,19]. In MOFs, metal framework centres and organic units link, thereby allowing structural transformations to occur upon the adsorption of gas [20,21,22,23,24,25] via rearrangement of the flexible linkers. Furthermore, temperature and pressure can also cause structural transformations to occur [24,26]. Coudert and co-workers [27] identify two successive transformations, from a large pore (LP) to a narrow pore (NP) state and back again to the LP state which they classify as guest-induced structural transformations termed breathing and opening of gates. These depend on applied conditions such as temperature and pressure [23,24,26], as well as adsorption [20,21,22,23,24,25] of gas molecules. Here we will show how pressure in particular can transform structures with large pores with important implications for gas storage for automobiles. 

Modelling these transformations [20,21,22,23,24,25] in MOFs is challenging and has attracted wide attention. During the past few years [28,29,30], we have developed several solvable pseudo one-dimensional statistical mechanical lattice theories of adsorption of gas on MOFs. Previously, we considered [29] a pseudo-one dimensional statistical mechanical theory of adsorption in a metal-organic framework (MOF) with both narrow and large pores which is solved exactly by a transfer matrix method in the Osmotic Ensemble (OE). More recently, [30] we considered a theory treated in the OE to describe pure component and mixture adsorption in MOFs in structures which can undergo pore transformation from narrow to large pores. The theory successfully describes the form of adsorption of gas isotherms in MOFs which reflect structural transformations induced by adsorption. 

Even when somewhat physically unrealistic, solvable statistical mechanical models have made a very significant contribution to developing the theory of condensed phases [31,32]. The model which we discuss below is not a chemically realistic description of a particular MOF but is a mathematically tractable theory of this material which displays all the expected characteristics of adsorption of gas in a MOF with large pores and allowing identification of the important features of this fundamentally and practically important problem. This work suggests the way forward for an extensive experimental and theoretical program using Monte-Carlo techniques to study the predictions made in this work. 

Remarkably, in addition to opening of gates and breathing in MOFs [27], which is the result of a transformation from a large pore (LP) to a narrow pore (NP) state, Krause et al. [33] recently observed NGA in the large-pore MOF DUT-49 which has a pore-system comprising an assembly of three pore-subsystems—cuboctahedral, tetrahedral and octahedral pores. Synchrotron powder X-ray diffraction studies during methane adsorption at 111 K have revealed a structural “twisting” transformation [34] of the large-pore structure DUT-49op at the pressure coinciding with the negative adsorption step of the isotherm. While the cuboctahedral pores size does not change, the size of the tetrahedral pores and especially the size of the octahedral pores decreases considerably. This phenomenon observed by Coudert and collaborators [33] seems to play a central role in large-pore MOFs for energy storage.

Usually, the adsorbed amount increases with gas pressure as the chemical potential of the adsorbed component in the gas phase increases. However, methane adsorption isotherms at low temperatures in DUT-49, a highly absorbent MOF, show a sudden decrease in the adsorbed amount with pressure at less than half of the full adsorption capacity of the adsorbent DUT-49 [33]. This phenomenon was successfully simulated using grand canonical Monte-Carlo methods [34] and appears rare amongst small pore MOFs. At low pressures, methane is adsorbed in the open pore system of DUT-49 that has high adsorption capacity. After a certain amount has been adsorbed, an adsorption-induced transformation takes place of the open pore system to a much lower capacity closed pore system. As the capacity of the closed pore system is lower than the adsorbed amount at the transformation instance, the excess amount is removed from the MOF structure, manifested as NGA which may be important technologically [35,36,37,38,39]. Most importantly, as we will discuss in detail below, we find two regions of NGA in MOFs with large pores. Our theoretical study suggests that NGA may actually be quite common in large pore MOFs and be a significant factor in the design of gas storage in MOFs for energy applications.

In this paper, we extend this work and present the results of a fundamental theoretical study of small molecule adsorption in a generic large pore MOF with the purpose of identifying features important in gas storage. Quite unexpected features occur which may have a very significant impact on the use of MOFs in gas storage. Thus, we extend our statistical mechanical theory to describe adsorption of single components in a generic large pore MOF as a theory for gas storage material. Of great interest for gas storage we find two regions of NGA where gas pressure causes collapse of the structure. These transformations can be driven by applied compressive stress and possibly utilised in an engine to release adsorbed gas from the MOF. The factors which govern this NGA are identified. We do not consider specific MOF structural details but focus on developing a statistical mechanical theory which mimics the essential features of NGA and make predictions about the adsorption behaviors of large pore MOFs.

## 2. Pseudo-One Dimensional Model of Large-Pore Metal-Organic Frameworks 

Some time ago, we proposed an exactly solvable transfer matrix treatment of a statistical mechanical lattice theory of a MOF which allows mixture and single component adsorption isotherms and the compression of these soft materials to be theoretically described [30]. There is a broadly held view [27] that the OE is the most appropriate theoretical formalism to model adsorption in soft porous materials. The OE was developed initially by Brennan and Madden [40] and Panagiotopoulos [41]. The theoretical approach taken here is an extension of our previous development of an exactly solvable statistical mechanical lattice theory of a MOF in the OE using a transfer matrix method which considers the treatments of the solid and gas components in an even handed way [28,30]. 

Coudert and co-workers [42,43] in particular developed the OE for molecular simulation of adsorption in MOFs. In his seminal text, Hill [44] has discussed a number of “Generalized Ensembles” and the OE belongs in this class. For the OE, the independent thermodynamic variables are temperature ***T***, the number unit cells *N* in the MOF, the compressive stress *σ* and the chemical potentials $\mu_{a}$ of the gas molecules *a.* The compressive stress *σ* and the chemical potential $\mu_{a}$ are assumed to be independent variables. 

Here we develop this transfer matrix treatment of a pseudo-one-dimensional statistical mechanical OE theory of pressure and adsorption-induced structural transformations in MOFs with large pores and in this way predict that NGA is common in such solids. The evaluation of the Osmotic Potential requires the solution of a matrix eigenvalue problem which may be treated computationally or exactly in some cases. It is found that for weak unit-cell interactions we can calculate all the eigenvalues of the OE transfer matrix analytically. 

**Figure 1:**
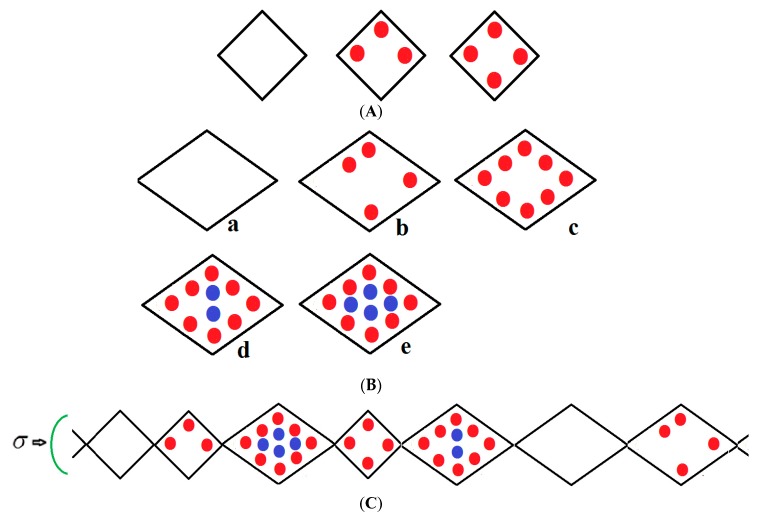
(**A**) Vacant, partially filled and full narrow pore; (**B**) Large pore, (a) vacant, (b) partially filled first layer, (c) filled first layer, (d) filled first layer and partially filled second layer (e), fully filled large pore.; (**C**) An example of a typical arrangement of a cluster of molecules considered in the pseudo-one-dimensional chain.

We consider a chain of *N* groups of unit cells each, which may be in either a narrow pore (NP) or large pore (LP) state with volumes $v_{\mathrm{NP}}\;\mathrm{and}\;v_{\mathrm{LP}}$; the NP and LP volumes which are variable but are typically 5000 Å^3^, 10,000 Å^3^ ,respectively, yield a 50% volume difference between these two states. The chain runs along the *x*-direction and is compressed by mechanical compressive stress *σ* (loosely termed pressure) parallel with this axis as shown in Figure 1. If no external stress is applied, the mechanical stress is equal to the gas pressure *P* but otherwise *σ* and *P* are independent variables. The stress term *σ* includes the gas pressure *P* and any applied compressive stress. The NP can be filled by up to a monolayer of gas molecules as depicted in Figure 1A. However, the LP can have adsorbed multilayers as shown in Figure 1B. Figure 1C shows an example of a typical configuration of a group of species considered in the infinite pseudo-one dimensional chain. The structure is subjected to a mechanical compressive stress *σ* (pressure) directed along the *x*-axis. The NP on the far left of Figure 1C is vacant while the next small pore contains three methane molecules in the first adsorbed layer (red); the next LP to the right contains eight molecules in the first layer and four (blue) in the second layer etc. Molecules are treated as spheres. All energetically allowed configurations and cell occupations are taken into consideration. The numbers of molecules in the NP and LP are variables in the theory. In Figure 1A–C, all molecules in the first monolayer are shown in red and blue in the second layer. In reality, much larger NPs and LPs are considered but these are difficult to display.

The above description is clearly limited but it is a theory which straightforwardly yields predictions of the compressive and adsorption properties of MOFs which are difficult and expensive to obtain otherwise. This approach is not a substitute for more demanding computer simulations of three dimensional MOFs nor do we expect the empirical parameters found for our pseudo-one dimensional theory to realistically describe a real MOF. Compared with simulation-based approaches, our aims and objectives differ somewhat. Accurately treated statistical mechanical models may easily give original predictions of novel behavior in MOFs. Hence, our purpose is to construct a methodology which enables the broad features of NGA isotherms to be calculated easily and cheaply and which may give up new insights into these materials. 

All energetically allowed occupations and configurations are permitted in the theory. The chain is compressed by a stress σ (pressure) directed along the *x*-axis. The small pore (NP) on the extreme left is vacant while the next small pore contains three methane molecules in the first adsorbed layer (red); the next LP to the right contains eight molecules in the first layer and four (blue) in the second layer etc. Molecules are treated as spheres. The numbers of molecules in the NP and LP are variables in the theory.

In all figures, molecules in the first monolayer are shown in red and blue in the second layer. In reality, much larger NPs and LPs with many molecules are considered. However, these are difficult to display, but they are defined in the captions of the figures below.

The adsorbed species occupying the cells are in equilibrium with those in an ideal gas phase at pressure *P* and temperature *T* with chemical potential $\mu_{}=\mu^{0}+\;kT\ln(P)$ where the standard chemical potential $\mu^{0}$ is given by $\mu^{0}=-kT\ln\left[{\left(\frac{2\pi mkT}{h^{2}}\right)}^{3/2}kT\right]$ and where non-ideal behaviour can be introduced substituting pressure by fugacity, see Hill [45]. m is the molecular mass, *k* is Boltzmann’s constant and *h* is Planck’s constant.

For species adsorbed in the one-dimensional chain of *N* cells, the Osmotic Partition function Φ(σ,T,μ) is:

(1) $$\Phi (\sigma ,T,\mu )=\sum_{V}\sum_{n}\exp(-\frac{\sigma V}{kT})Q(n,V,T)\exp(\frac{\mu n_{}}{kT})$$

Q(n,V,T) is the Canonical partition function for volume *V*. The Osmotic Potential Ω is Ω=−kTlnΦ calculated in this application from the logarithm of the maximum term in the Osmotic partition function. Here, we evaluate this accurately by a transfer matrix method and hence compute adsorption isotherms.

Hence, the logarithm of the maximum term in the series Equation (1) is:

(2) $$\ln\Phi =-\frac{\sigma V^{*}}{kT}+\ln Q(n^{*},V^{*},T)+\frac{\mu n^{*}}{kT}$$

The starred (*) symbol indicates optimum values $n^{*},V^{*}$ which are required to be solutions to the extremum Equations

(3) $$(\frac{\partial \ln\Phi }{\partial V})=\frac{-\sigma }{kT}+(\frac{\partial \ln Q}{\partial V})=0\;(\frac{\partial \ln\Phi }{\partial n})=(\frac{\partial \ln Q}{\partial n_{}})+\frac{\mu }{kT}=0$$

These expressions are identical to those for pressure and chemical potential in a canonical ensemble, demonstrating that the use of Maximum term methodology has caused the OE to degenerate into a Canonical ensemble [45]. Differentiation of Equation (2) yields

(4) $$-d(kT\ln\Phi )=V^{*}d\sigma -SdT-n*d\mu \;$$

giving for the optimum values $n^{*},V^{*}$:

(5) $$V^{*}=-kT(\frac{\partial \ln\Phi }{\partial \sigma })\;n^{*}=kT(\frac{\partial \ln\Phi }{\partial \mu })$$

lnΦ is obtained using the transfer matrix method described in the next section. 

## 3. Transfer Matrix Method for Calculation of Adsorption Isotherms for Large-Pore Metal-Organic Frameworks.

We have previously given a review of matrix methods for the calculation of adsorption isotherms for one-dimensional lattice fluids [46]. A recent application of this approach to MOFs has been given by Simon et al. [47].

The Osmotic partition function Equation (1) can be expressed as [29]:

(6) $$\Phi =\sum_{\alpha =1}^{j}\sum_{\beta =1}^{j}\sum_{\gamma =1}^{j}\ldots \sum_{\omega =1}^{j}A_{\alpha \beta }A_{\beta \gamma }A_{\gamma \delta }\ldots A_{\omega \alpha }$$

By wrapping the chain on to a ring, cyclic boundary conditions will be imposed. In the matrix formalism, we specify the terms A*_αβ_* in (6) as the multiple of the internal partition functions *f_α_* for cluster *α* and *f_β_* for cluster *β* and an intercluster interaction expression given by:

(7) $$A_{\alpha \beta }={\left(f_{\alpha }f_{\beta }\right)}^{1/2}e^{-\varepsilon_{\alpha \beta }/kT}$$

The variables *α*, *β* span all clusters 1 to *j* and where *ε_αβ_* is the interaction energy between these.

We have noticed that the important features of the MOF adsorption isotherms can be obtained by considering two extreme cases by choosing particular values of *ε_αβ_*. This parameter is set to zero or infinitely repulsive and this choice defines theories A and B below. 

Using standard matrix algebra we have $D_{\mathrm{ij}}=\sum_{k}B_{\mathrm{ik}}C_{\mathrm{kj}}$ for the inner matrix product of a pair of conformable matrices **B** and **C.** Hence, the Osmotic Partition function (6) is given as

(8) $$\Phi (\sigma ,T,\mu_{a},\mu_{b})=\sum_{\alpha =1}^{j}{\left(A^{N}\right)}_{\alpha \alpha }\;=\;\mathrm{Tr}\;(A^{N})\;=\;\sum_{i=1}^{j}{\left(\lambda_{\;i}\right)}^{N}$$

The transfer matrix A has elements

(9) $$A_{ij}={\left(f_{i}f_{j}\right)}^{1/2}e^{-\varepsilon_{ij}/kT}$$

and has eigenvalues $\lambda_{1},\;\lambda_{2},\;\lambda_{3},\ldots \;\lambda_{j}$. Only the largest eigenvalue $\lambda_{\max}$ of **A** is required in matrix evaluations of partition functions, since for large *N,* Equation (6) reduces to

(10) $$\Phi (\sigma ,T,\mu )={\left(\lambda_{\max}\right)}^{N}$$

Δ is the energy cost to transform the more stable LP conformer to the NP conformation. We will assume in this minimum theory that the narrow pore only has a single layer. 

For $n_{1}$ molecules absorbed in the first monolayer of the LP we assume that a cluster of molecules and vacancies occupy $N_{\max LP}$ sites. With $n_{1}$ molecules there are $\left(N_{\max LP}-n_{1}\right)$ holes or vacancies giving rise to a configurational degeneracy (the first factor on the right hand side in Equation (11)). 

For a cluster containing *n* molecules, the number of pair interactions is estimated as $(n^{2}-n)/2$ If the mean interaction energy is *J*, the total interaction energy is $J(n^{2}-n)/2$. 

Thus, the cluster partition function $f_{LP,n_{1}}$ for the first monolayer of molecules in the LP containing $n_{1}$ molecules is

(11) $$f_{LP,n_{1}}=\frac{N_{\max LP_{1}}!}{(N_{\max LP_{1}}-n_{1})!n_{1}!}\exp(\frac{-\sigma \upsilon_{LP}}{kT}){\left(\exp(\frac{-u_{LP_{1}}+\mu }{kT})\right)}^{n_{1}^{}}\exp(\frac{-J(n_{1}{}^{2}-n_{1})/2}{kT})$$

where $u_{LP_{1}}$ is the adsorption energy of a molecule in the first monolayer in the LP.

Using similar arguments, a NP cell containing n1 species makes a contribution to the Osmotic partition function

(12) $$f_{NP,n_{1}}=\frac{N_{\max NP_{1}^{}}!}{(N_{\max NP_{1}}-n_{1})!n_{1}!}\exp(\frac{-\sigma \upsilon_{NP}-\Delta }{kT}){\left(\exp(\frac{-u_{NP_{1}^{}}+\mu }{kT})\right)}^{n_{1}}\exp(\frac{-J(n_{1}{}^{2}-n_{1})/2}{kT})$$

After the first layer in the LP is filled, multilayer adsorption can occur. The large pore has molecules adsorbed in other layers which are not tightly bound. The partition function for these multilayer species containing Nm molecules is given by

(13) $$\begin{array}{l} f_{LP,N_{m}}=\frac{N_{outer}!}{(N_{outer}-N_{m})!N_{m}!}\exp(\frac{-\sigma \upsilon_{LP}}{kT}){\left(\exp(\frac{-u_{outer}+\mu }{kT})\right)}^{N_{m}}\exp(\frac{-J(N_{m}{}^{2}-N_{m})/2}{kT})\\ \times \exp(\frac{-u_{LP}+\mu }{kT}){)}^{N_{\max LP}}\exp(\frac{-J(N_{\max LP}{}^{2}-N_{\max LP})/2}{kT}) \end{array}$$

$N_{outer}$ is the number of sites in the outer layer, and the energy of adsorption $u_{outer}$ is scaled (by a factor ‘scale’ in the figure captions below) to give a reduction factor in the well-depth and also takes account phenomenologically of the interlayer interactions.

## 4. Eigenvalues of the Transfer Matrix

To calculate adsorption isotherms, the largest eigenvalue of the transfer matrix given in Equation (9) is needed and usually this must be obtained by computational means. However, we have studied two particular relevant cases where it is possible to find all the eigenvalues of the Transfer Matrix algebraically by exploiting elements of the theory of symmetrical matrices. For the two cases with the block structures shown in Equations (15) and (16) below, it is demonstrated in Reference [30] that the largest eigenvalue of the appropriate block is

(14) $$\lambda_{\max}=(\sum_{i=1}^{j}f_{i})$$

and all the other eigenvalues are zero. 

The matrix A (defined by Equation (9)) can be partitioned into two main blocks which describe LP and NP cell types and off-diagonal blocks which implement the coupling between these two types of conformations as indicated below

(15) $$\left(\begin{array}{cc} A_{LP,LP} & A_{LP,NP}\\ A_{NP,LP} & A_{NP,NP} \end{array}\right)$$

The coupling (or otherwise) of the main diagonal blocks by the off-diagonal blocks gives two types of theories—A and B.

Coudert and co-workers [48] assume that LP and NP phases do not exist simultaneously in a perfect crystal. In such a situation, off-diagonal couplings are zero giving the matrix

(16) $$\left(\begin{array}{cc} A_{LP,LP} & 0\\ 0 & A_{NP,NP} \end{array}\right)$$

The main diagonal blocks in this matrix decouple and this permits calculations to be made for ordered crystals, which we call theory A in Reference [30], and here “sharp transition theory”.

Introduction of this off-diagonal coupling permits consideration of short range ordered phase mixtures which we call theory B as in Reference [30], and here “gradual transformation theory”.

Below, we present some results for both theories as appropriate to the level of disorder in the MOF sample.

## 5. Effect of Compressive Stress on a MOF

**Figure 2:**
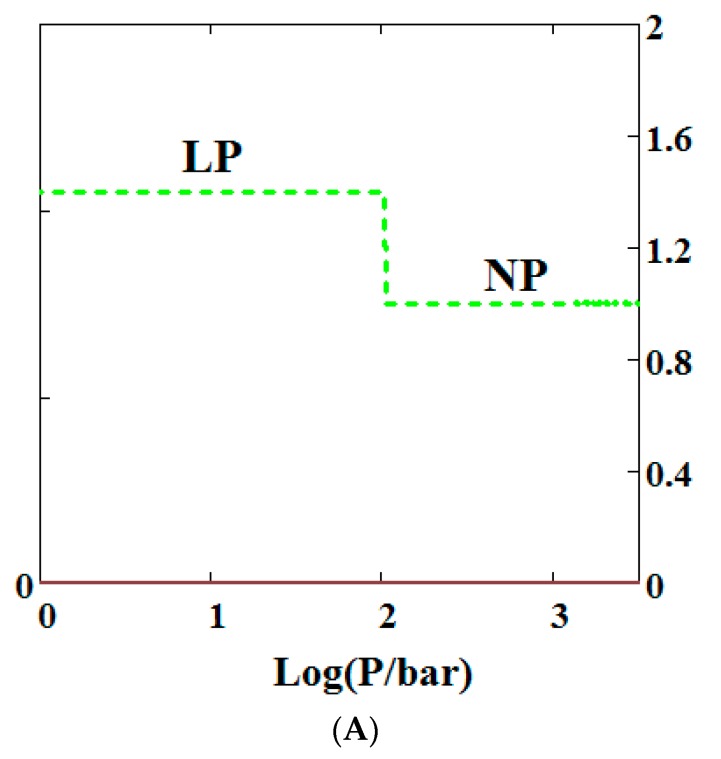

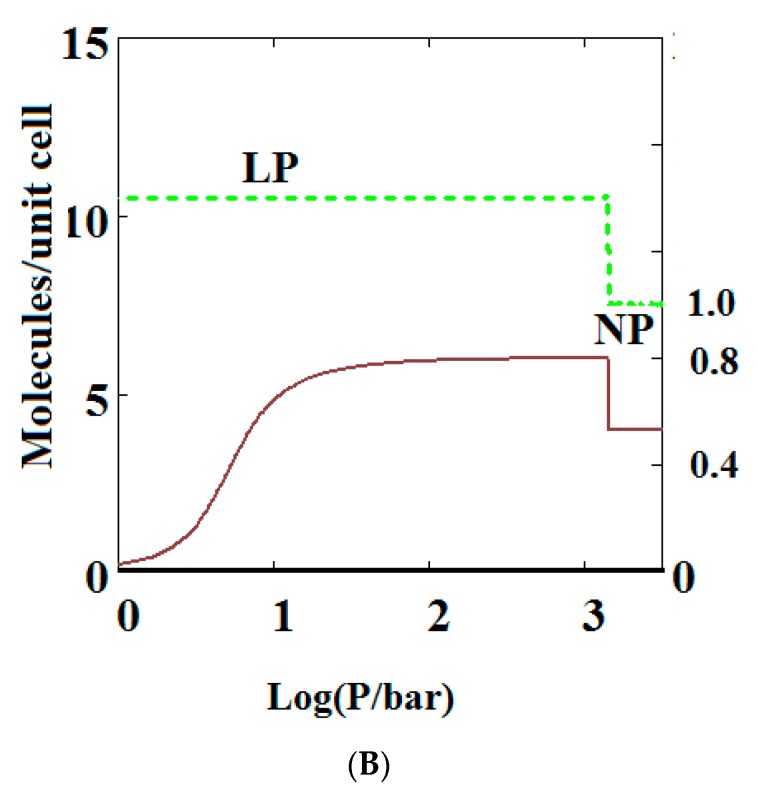
(**A**) Mechanical compression of MIL-53 at 300 K, using the parameters given in Reference [30]. At higher pressures there is mechanical compression of the metal-organic frameworks (MOF) to the narrow pore (NP) state. The axis on the right measures the volume in units of the NP volume; (**B**) adsorption isotherms of pure methane calculated using a sharp transformation approach at 300 K using the pure methane parameters given in Reference [30] for MIL-53. At higher pressures there is negative gas adsorption (NGA) caused by the compression of the MOF to the NP state. The axis on the right measures the solid volume in units of the NP volume.

Mercury intrusion experiments have been undertaken by Beurroies et al. [49] and Yot et al. [50] in which a powdered sample of the MOF MIL-53 is brought under isotropic mechanical stress causing the powder to increase in density. Such a mechanical stress corresponds crudely to compressive stress (loosely termed a mechanical pressure σ in our theory). Later, Neimark et al. [22,23,24] observed a LP to NP transformation at a pressure of 550 ± 150 bar. We have modelled this compression-induced density change by calculating the solid volume as a function of the mechanical compression of a theory of MIL-53 parameterised as in Reference [30] to give a good description of pure methane adsorption isotherms. Figure 2A below shows compression of the structure, which is in reasonable agreement with the results in the above studies. The same theory was used and Figure 2B shows an adsorption isotherm calculated for pure methane for MIL-53. The adsorption isotherm shows NGA at about 1000 bar which is about the same mechanical pressure as the intrusion experiments undertaken by Beurroies et al. [49] while Neimark et al. [24] showed collapse of the MIL-53 structure when brought under isotropic mechanical stress, causing the powder to increase in density. The pressure at which NGA occurs increases in the model with increasing heat of adsorption, indicating that it is harder to compress the MOF containing more strongly bound molecules. As the temperature is lowered to below 284 K, our calculations also show that MIL-53 breathes in pure methane where the NP plays a significant role [30].

## 6. Isotherms Showing Negative Adsorption of Gas at Low Pressure

**Figure 3:**
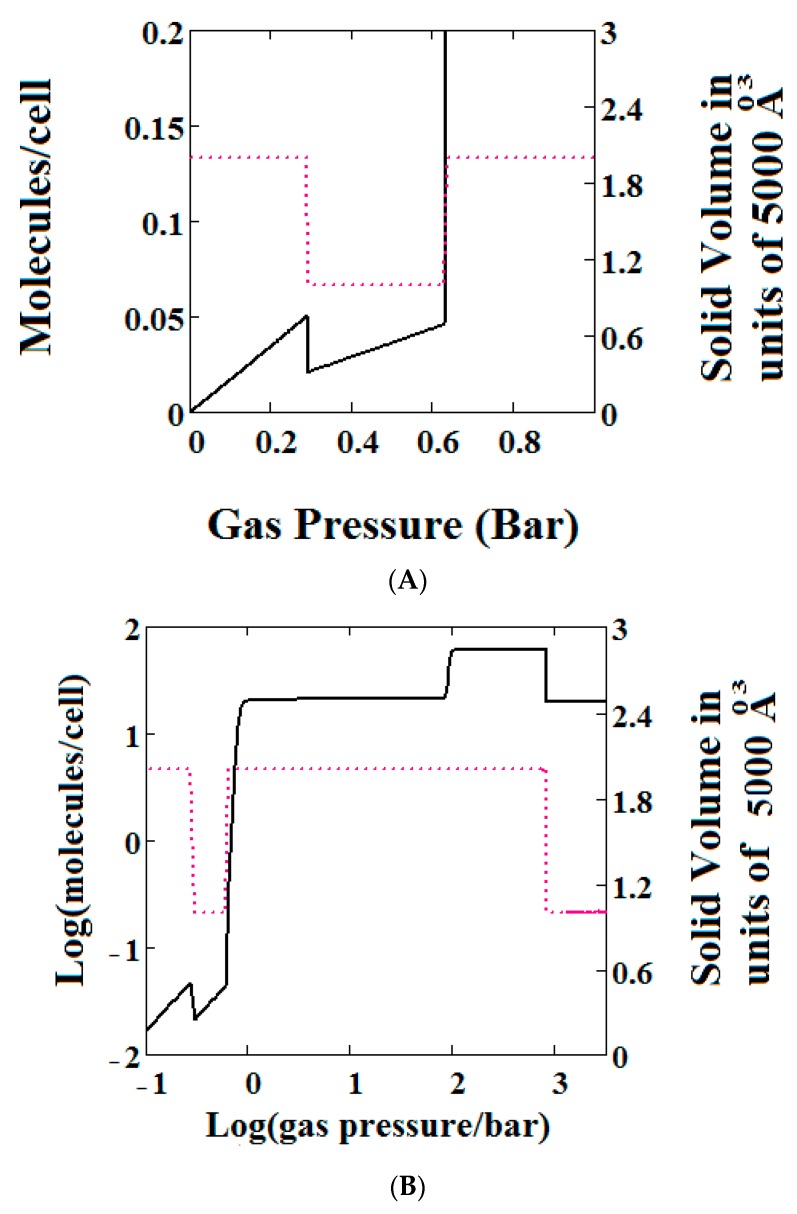
(**A**) Methane adsorption in the theory MOF showing Negative gas adsorption (NGA). The dotted red curve on the secondary right axis indicates the (narrow pore/large pore) NP/LP state of the MOF. The other parameters are *T* = 257 K, uglp = −14.8 kJ/mole, ugnp = −13.55 kJ/mole, *J* = −1.08 kJ/mole, *X* = 5000 Å^3^, *Y* = 10,000Å^3^, Δ = 0.025 kJ/mole, *Nm* = 40, scale = 0, nmaxlp = 21, nmaxnpb = 20; (**B**) same methane adsorption in the theory MOF on a Log-Log scale showing 2 regions of NGA.

**Figure 4:**
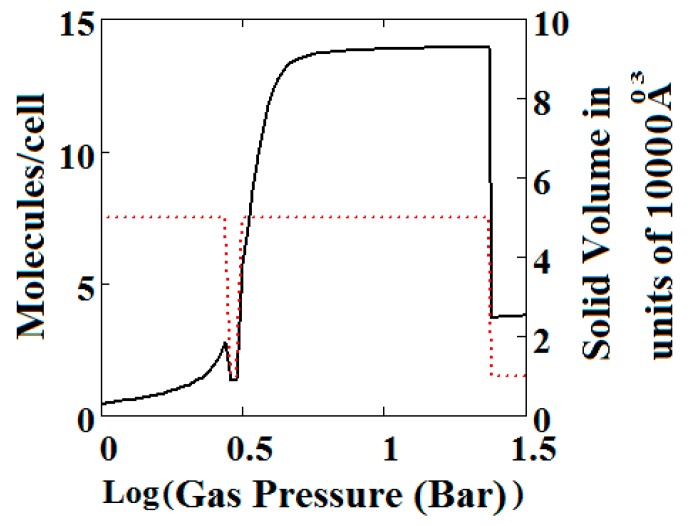
Methane adsorption in the theory MOF showing two regions of NGA. The dotted red curve on the secondary right axis indicates the NP/LP state of the MOF. The other parameters are T = 285 K, uglp = −29.1 kJ/mole, ugnp = −28.69 kJ/mole, *J* = −1.66 kJ/mole, Δ = 5.8 kJ/mole, *X* = 10,000 Å^3^, *Y* = 50,000 Å^3^. At high gas pressure the MOF is compressed into the NP state. The behaviour shown at high pressure should be almost universal for MOFs. Nm = 10, scale = 0.85, nmaxlp = 4, nmaxnpb = 4 (sharp transformation theory).

**Figure 5:**
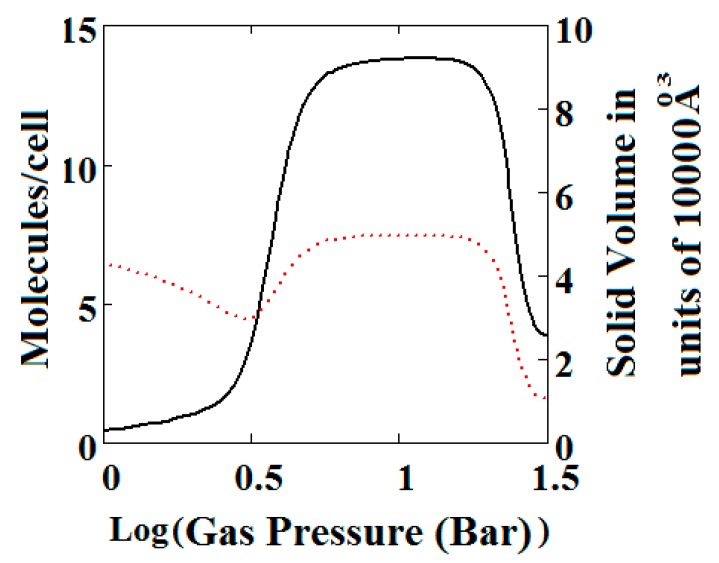
Plot of the behaviour of the methane adsorption in the theory MOF showing two regions of NGA transformation. The dotted red curve on the secondary right axis indicates the NP/LP state of the MOF. The other parameters are *T* = 285 K, uglp = −29.1 kJ/mole, ugnp = −28.69 kJ/mole, J = −1.66 kJ/mole, Δ = 5.8 kJ/mole, *X* = 10,000 Å^3^, *Y* = 50,000 Å^3^. At high gas pressure the MOF is compressed into the NP state. The behaviour shown at high pressure should be almost universal for MOFs. *Nm* = 10, scale = 0.85, nmaxlp = 4, nmaxnpb = 4 (gradual transformation theory). Compare with Figure 4.

Adsorption isotherms have been calculated and presented in Figure 3, Figure 4 and Figure 5 using the parameters shown below the figures. The energies of adsorption of the pure components and maximum occupations for the large (two types of site) and narrow pores are parameterized and given in the figure captions. Δ is the energy difference between the LP and NP states and is the energy required to convert from the more stable LP to NP.

Calculation of MOF volumes and adsorption isotherms was performed straightforwardly using the above methodology and the Mathcad 15 software package [51]. Finite difference calculation of derivatives was performed. 

The methane adsorption isotherm in Figure 3A shows a negative adsorption at around 0.3 bar which is the result of the LP to NP transformation at the same pressure. The vacant MOF is in the LP state. If we consider each pore system on their own, LP or NP, having their individual isotherms [48], then the resulting isotherm can be considered to be constructed from the segments of the individual isotherms of the stable pore system. Usually, in the low pressure region, the LP isotherm lies below the NP isotherm [48]. As a result, when the LP to NP transformation occurs, the adsorption isotherm shows a positive step, as normal. Under some conditions, such as those in the experimental work [33] and our calculations presented here, the LP-NP isotherms at low pressure reverse. Under these conditions, the LP isotherm lies above the NP. Hence, the LP to NP transformation causes a negative adsorption step.

Figure 3B, which is a log-log plot of the same isotherm and extends to much higher pressures, shows a second negative adsorption just below 1000 bar, when the NP system is transformed back to the LP one. This adsorption step is negative because at those pressures, LP has higher adsorption capacity than NP. Before this, at about 100 bar, the isotherm shows a positive adsorption step in the LP state. Up to this step, the adsorption takes place in the first adsorption monolayer which is saturated quickly after the NP to LP transformation at 0.3 bar, while at around 100 bar, the inner core of the LP theory, the second layer, fills rapidly. This behaviour arises for a wide range of theory parameters such as the ones in Figure 4. 

Similar behaviour is also shown by the gradual transformation theory [30] in Figure 5. Rather than sharp adsorption steps, the MOF structure shows a gradual negative adsorption at the pressure range 20–30 bar. In the gradual transformation theory, the coexistence of LPs and NPs is permitted. As the solid volume curve in Figure 5 shows, at 1 bar a mixture of NPs and LPs coexist. With increasing pressure, LPs are transformed to NPs. This transformation is complete at around 5 bar, where both curves level the adsorption isotherm as well as the solid volume to the LP volume value. At around 20 bar, the reverse transformation of LPs to NPs starts taking place which causes a gradual negative adsorption. In Figure 5, the first negative adsorption transformation shown in Figure 4 is lost.

## 7. Influence of Mechanical Pressure on Adsorption Isotherms 

**Figure 6:**
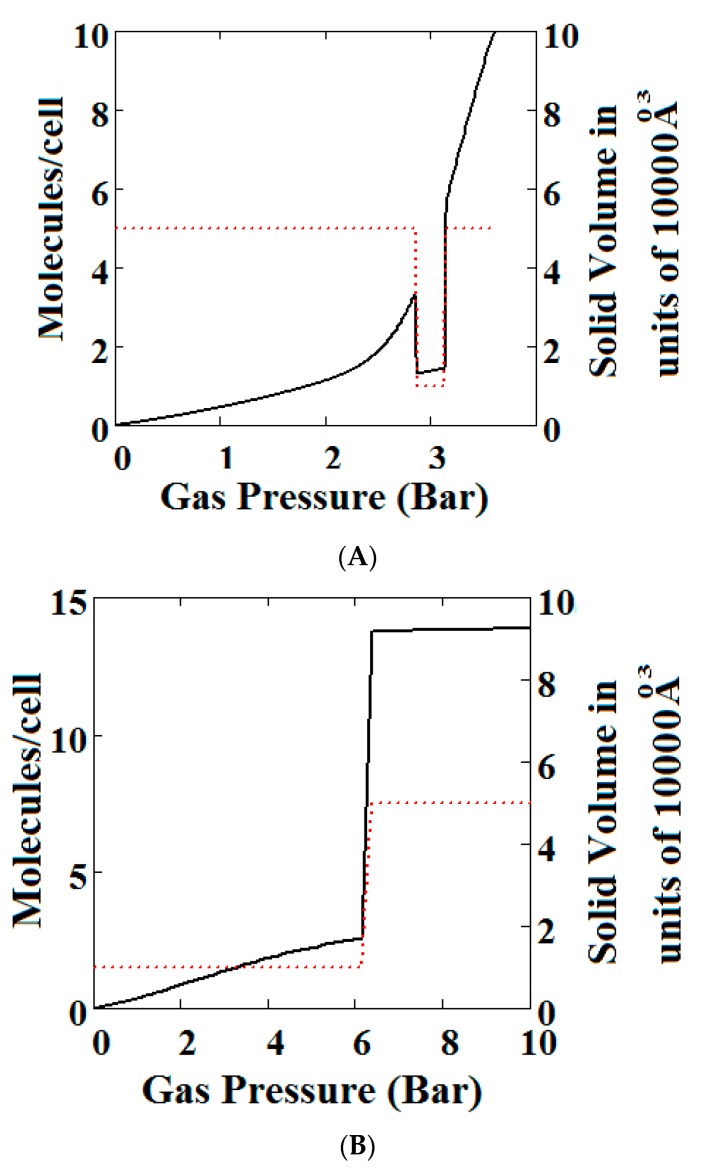

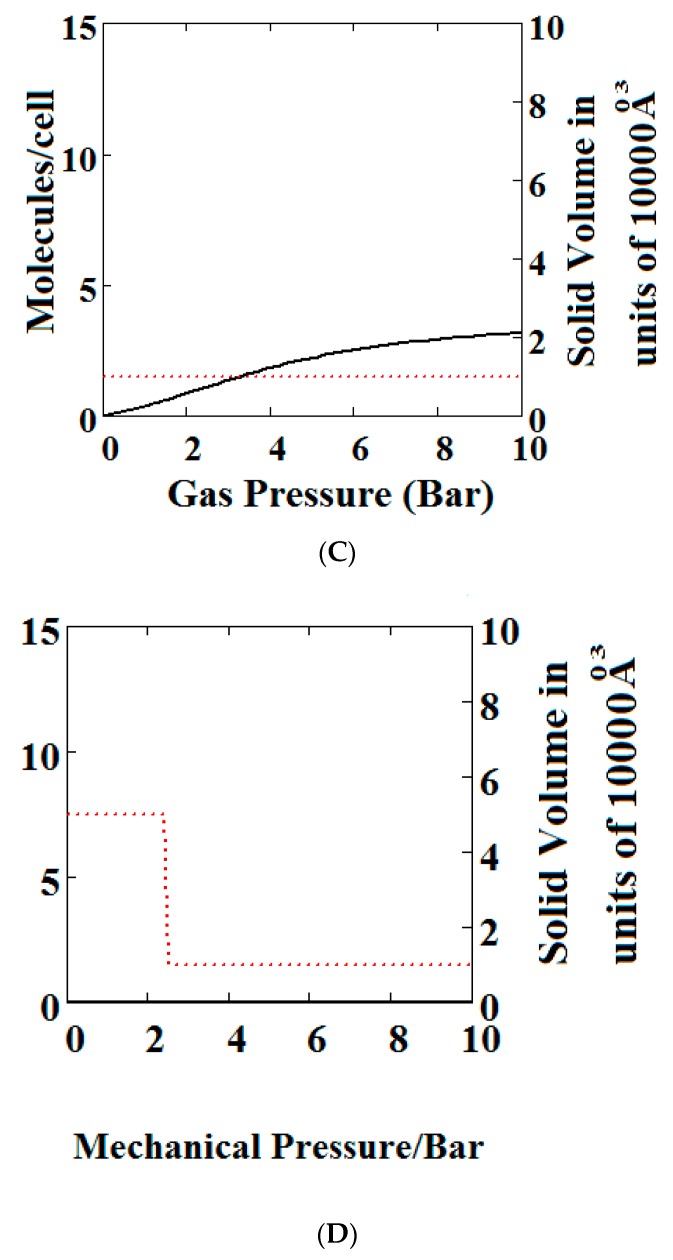
Plot of the behaviour of the methane adsorption in the theory MOF under an additional applied mechanical pressure of (**A**) 0 Bar; (**B**) 10 Bar; (**C**) 100 Bar. In (**B**,**C**) the NGA transformation shown in (**A**) has disappeared. The dotted red curve on the secondary right axis indicates the NP/LP state of the MOF; (**D**) Plot of the density behaviour of the MOF under an applied compressive stress. The dotted red curve on the secondary right axis indicates the NP/LP state of the MOF. The other parameters are *T* = 285 K, uglp = −29.1 kJ/mole, ugnp = −28.69 kJ/mole, *J* = −1.66 kJ/mole, Δ = 5.8 kJ/mole, *X* = 10,000 Å^3^, *Y* = 50,000 Å^3^, *Nm* = 10, scale = 0.85, nmaxlp = 4, nmaxnpb = 4.

We have calculated using Theory A (sharp transformation approach) adsorption isotherms (Figure 6) when the MOF structure is subjected to various additional compressive stresses over a range of gas phase pressures as shown in Figure 6. In Figure 6A, no additional compressive stress is applied, while in Figure 6B,C additional mechanical pressures of 10 and 100 bar respectively are applied. It may be seen that the negative adsorption disappears in Figure 6B,C with the application of additional compressive stress. Under 10 bar (Figure 6B) additional compressive stress, at low gas pressures, the MOF is in the NP state transforming to the LP at higher gas pressures, while at 100 bar (Figure 6C) the transformation does not occur at all, the system remains in the NP state for the pressure range considered. The application of additional compressive stress squeezes the MOF structure into the NP state, such that in all cases the system remains in the NP state.

In Figure 6D, we show the effect of increasing mechanical pressure on the density of the MOF in the absence of gas pressure. It can be seen that the system is in the LP state at low compression and collapses to the NP state at about 2.5 bar. This pressure is close to the gas pressure shown in Figure 6A at which the MOF shows NGA. 

When adsorbents are used for gas storage, slow release of the gas is problematic. It seems possible that in an MOF with slow desorption kinetics being used for gas storage, application of mechanical compression might speed up the release of gas. 

## 8. Effect of Various Parameters on NGA: Intermolecular Interactions, Heat of Adsorption, Pore Transformation Energy

**Figure 7:**
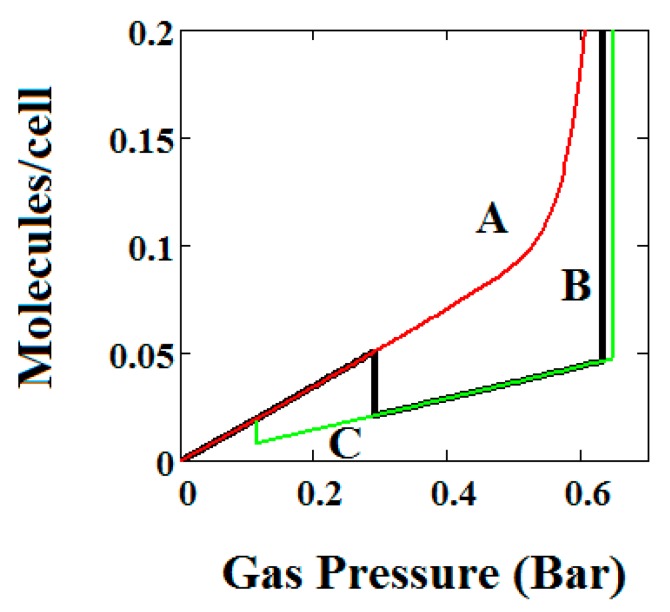
Plot of the behaviour of the methane adsorption in the theory MOF for three values of the parameter Δ (A = 0.05, B = 0.025, C = 0.01 kJ/mole). Curves B and C show a NGA transformation. Low values of Δ shift the NGA transformation to lower pressures while high values cause the transformation to disappear. The other parameters are *T* = 257 K, uglp = −14.8 kJ/mole, ugnp = −13.55 kJ/mole, *J* = −1.08 kJ/mole, *X* = 5000 Å^3^, *Y* = 10,000 Å^3^, Nm = 40, scale = 0, nmaxlp = 21, nmaxnpb = 20.

**Figure 8:**
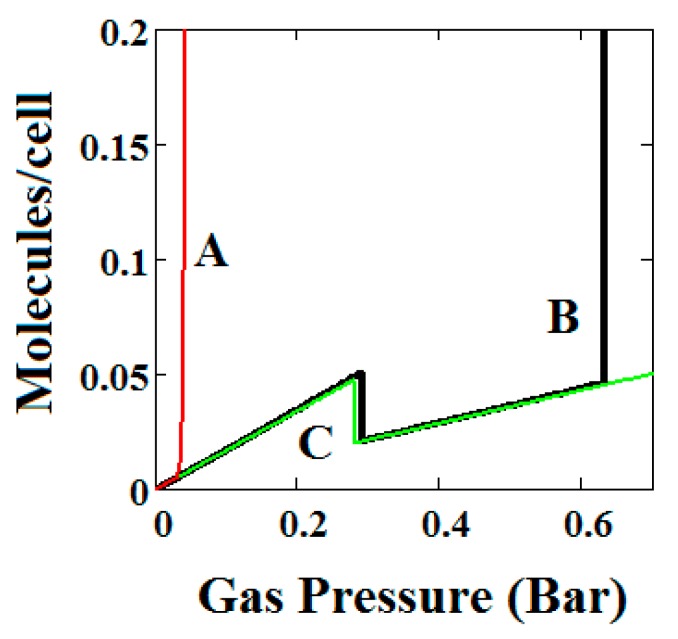
Plot of the behaviour of the methane adsorption in the theory MOF for three values of the parameter *J* (*A* = −2.29, *B* = −1.49, *C* = −0.573 kJ/mole). Curves B and C show NGA transformation. High J values cause the NGA transformation to disappear. The other parameters are *T* = 257 K, uglp = −14.8 kJ/mole, ugnp = −13.55 kJ/mole, Δ = 0.025 kJ/mole, *X* = 5000 Å^3^, *Y* = 10,000 Å^3^, *Nm* = 40, scale = 0, nmaxlp = 21, nmaxnpb = 20.

**Figure 9:**
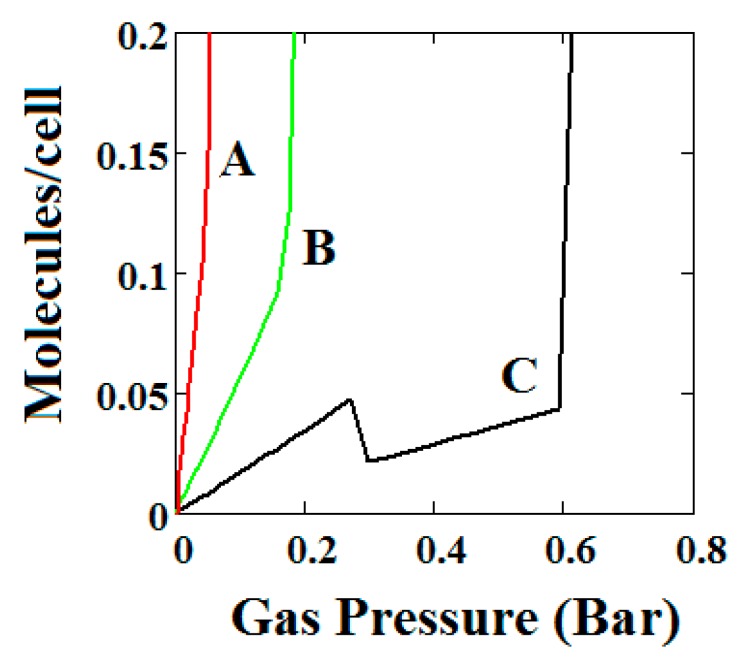
Plot of the behaviour of the methane adsorption in the theory MOF for three pairs of differences in values of the LP and NP heats of adsorption (*A* = −13.00/−18.96, *B* = −16.6/−13.55, *C* = −14.8/−13.55 kJ/mole). Curve C shows a NGA transformation at low pressure on the left. For the NGA transformation to occur, the LP, NP heats of adsorption should be close in magnitude. The other parameters are *T* = 257 K, *J* = −1.08 kJ/mole, Δ = 0.025 kJ/mole, *X* = 5000 Å^3^, *Y* = 10,000 Å^3^, *Nm* = 40, scale = 0, nmaxlp = 21, nmaxnpb = 20.

In Figure 2, Figure 3, Figure 4, Figure 5 and Figure 6, we have shown examples of NGA for a wide range of model parameters. Figure 7, Figure 8 and Figure 9 shows the effect of variation of the parameters representing pore transformation energy on the adsorption isotherms (Δ), intermolecular interactions (*J*) and differences in values of the LP and NP heats of adsorption.

The behaviours shown are consistent with the importance of these terms in the osmotic potential Ω given by Ω=−kTlnΦ where the system minimises this potential. Thus, in Figure 7, the system with the lowest value of Δ undergoes a negative adsorption transformation most readily, while in Figure 8, the system with the lowest value of J (intermolecular interactions in LP) undergoes a negative adsorption transformation most readily. Thus, low values of Δ shift the NGA transformation to lower pressures while high values cause the transformation to disappear; high J values cause the NGA transformation to disappear.

In Figure 9, isotherms are compared for three pairs of differences in values of the LP and NP heats of adsorption (A = −13.00 and −18.96, B = −16.6 and −13.55, C = −14.8 and −13.55, kJ/mole). Curve C shows an NGA transformation at low pressure on the left. For the NGA transformation to occur, the LP, NP heats of adsorption should be close in magnitude. 

## 9. Conclusions 

In this paper, we have presented the outcomes of a basic theoretical investigation of methane adsorption in large pore MOFs with the aim of establishing the unique features of this phenomenon relevant to the question of methane storage for energy applications. We have developed a pseudo-one-dimensional statistical mechanical theory of adsorption of gas in a MOF with both narrow and large pores which is solved exactly using a transfer matrix technique in the Osmotic Ensemble (OE). The theory effectively allows the distinctive features of adsorption of gas isotherms in MOFs to be described. The characteristic form of adsorption isotherms in MOFs reflect changes in structure caused by adsorption of gas and compressive stress. Of extraordinary importance for gas storage for energy applications we find two regimes of Negative adsorption of gas (NGA) where gas pressure causes the MOF to transform from the large pore to the narrow pore structure. These transformations can be induced by mechanical compression and conceivably used in an engine to discharge adsorbed gas from the MOF. The elements which govern NGA in MOFs with large pores are identified. 

Although it might appear that some of our conclusions are unfavourable when it comes to using MOFs in energy storage, this is not the case. For example, the negative gas adsorption phenomena which we discuss can be advantageously used in the release of gas from an MOF. There seems to be numerous possibilities around this issue. 

In certain situations, the NGA phenomenon may prove to be disadvantageous. However, it seems possible by use of chemical design to arrange the crystal energetics to eliminate this phenomenon so that the narrow pore plays no effective role in the adsorption. 

We have given no discussion to mixture adsorption [30] in these large pore MOFs. Yet, again there are many interesting questions and possibilities surrounding this issue.

Finally, we fully appreciate that our study, which to some extent is preliminary, raises many theoretical and experimental questions around the use of MOFs as energy storage materials.
